# Efficacy and safety of eribulin mesylate in patients with locally advanced or metastatic breast cancer previously treated with anthracycline/taxanes

**DOI:** 10.1002/cam4.7295

**Published:** 2024-05-24

**Authors:** Lan Chen, Xi Yan, Ting Luo, Tinglun Tian, Ping He, Xiaorong Zhong

**Affiliations:** ^1^ Department of Medical Oncology, West China Hospital Sichuan University Chengdu Sichuan Province People's Republic of China; ^2^ Department of Medical Oncology Head and Neck Cancer Department, West China Hospital Chengdu Sichuan Province People's Republic of China

**Keywords:** advanced breast cancer, eribulin mesylate, observation study, safety, toxicity

## Abstract

**Background:**

This prospective real‐world study aimed to assess the efficacy and safety of eribulin in the clinical practice against advanced breast cancer (ABC) in China.

**Patients and Methods:**

In this study, eligible patients with inoperable locally advanced or metastatic breast cancer who had experienced prior neo−/adjuvant or failed the palliative treatment with anthracycline/taxanes were included. Eribulin (1.4 mg/m^2^) was infused intravenously on Day 1 and Day 8 every 3 weeks until disease progression or intolerable toxicity occurred. The progression‐free survival (PFS), overall response rate (ORR), disease control rate (DCR), and safety of the treatment were assessed.

**Results:**

One hundred and thirty‐four patients were enrolled. The median PFS (mPFS) was 4.3 months (95% CI: 0.3–15.4). The ORR and DCR was 32.1% and 79.1%, respectively. The mPFS of patients who received eribulin as first‐ or second‐line treatment was significantly better than those who received eribulin as ≥3‐line treatment (6.9 months [95% CI: 3.2–8.8] vs. 4.0 months [95% CI: 3.4–4.6], *p* = 0.006). The mPFS of patients with triple‐negative, HER2‐positive, and HER2(−)/HR(+) was 3.4 (95% CI: 2.7–4.1), 6.2 (95% CI: 2.3–10.1) and 5.0 months (95% CI: 4.1–5.9), respectively. HER2(+) patients had significantly longer PFS than TNBC patients (*p* = 0.022). Patients received combination therapy had a significantly longer mPFS than those who received eribulin monotherapy (5.0 months [95% CI 3.6–6.3] vs. 4.0 months [95% CI: 3.3–4.7] [*p* = 0.016]). Multivariate analysis revealed that MBC patients with a molecular typing of non‐TNBC receiving eribulin as ≤2‐line therapy and combination therapy had a low risk of disease progression. Neutropenia (33.58%), leukopenia (11.94%), and thrombocytopenia (4.48%) were the most common treatment‐related adverse events.

**Conclusion:**

Eribulin demonstrated effective clinical activity and a favorable tolerability profile in Chinese patients with ABC in the real‐world. The efficacy and safety profile were consistent with those reported in previous randomized phase 3 trials.

## 1 INTRODUCTION

Female breast cancer is the most commonly diagnosed cancer in 2020, causing about 0.68 million new cancer deaths worldwide and posing a serious threat to women's health.^1^ Roughly 8% of patients with newly diagnosed breast cancer have metastatic disease,^2^, ^3^ with 20%–30% eventually experiencing recurrence and/or metastasis.^4^, ^5^, ^6^ Despite advancements in the treatment, the 5‐year survival rate of breast cancer is only 26%.^7^ The overall survival (OS) is estimated at about 8–18 months in patients with triple‐negative breast cancer (TNBC), significantly lower than that of hormone receptor (HR)‐positive (2–3 years) patients.^8^, ^9^, ^10^, ^11^

Eribulin, a nontaxane microtubule dynamics inhibitor, has shown promise in improving the OS of breast cancer patients who had failed previous taxanes treatments. The EMBRACE study (Study‐305), involving 762 patients with locally recurrent/metastatic breast cancer (MBC), have demonstrated that eribulin treatment can extend OS by approximately 2.5 months compared with the physician's choice of treatment (median OS [mOS]: 13.1 vs. 10.6 months, HR = 0.81, *p* = 0.041).^12^ In Study‐301, the result showed that eribulin did not achieve a significant improvement in OS (mOS: 15.9 vs. 14.5 months; *p* = 0.056) compared with capecitabine in overall population. However, there was a significant survival benefit in TNBC patients, where eribulin significantly extend the mOS by 5 months and reduced the risk of death by 29.8%.^13^ Based on the findings of these studies, eribulin has been approved as a subsequent‐line treatment for MBC.

Eribulin, as a non‐paclitaxel microtubule dynamic inhibitor, offers therapeutic benefits in paclitaxel‐resistant patients by binding to high‐affinity sites distinct from taxanes. This binding leads to the formation of non‐functional protein polymers, irreversible mitosis blocking, and cell death.^14^ Moreover, eribulin regulates the tumor microenvironment through vascular remodeling, enhancement of the immune microenvironment, and reversal of the epithelial‐mesenchymal transformation.^15^, ^16^, ^17^ The widespread utilization of eribulin in clinical practice, along with previous studies highlighting its value in combination therapy for MBC,^18^, ^19^, ^20^, ^21^ underscores the need to extend the conclusions from clinical trials, which often exclude certain patient populations, to real‐world settings. This prospective real‐world study was conducted to evaluate the efficacy and safety of eribulin in Chinese patients with ABC in routine clinical practice, providing a valuable reference for the clinical application of eribulin real‐world settings.

### 1.1 Patients and treatment

This study was a prospective, non‐randomized, observational trial. Patients aged between 18 and 75 years with inoperable locally advanced or MBC were eligible. Patients had previously undergone prior neo−/adjuvant or palliative treatment with anthracycline/taxanes and experienced disease progression. Other key inclusion criteria included the presence of at least one measurable lesion, ECOG PS 0 ~ 2, and sufficient organ reserve function with no obvious treatment contraindications.

Eribulin (1.4 mg/m^2^) was infused intravenously on Day 1 and Day 8 every 3 weeks until disease progression (PD) or intolerable adverse reactions. Imaging evaluation such as computed tomography or magnetic resonance imaging scans was performed every two cycles, Blood analyses and physical examinations were conducted before administration of eribulin on Days 1 and 8 of each cycle. As an observational study, investigators had the discretion to decide whether or not to combine other drugs. Systemic treatment initiated beyond 12 months after neo‐/adjuvant chemotherapy was defined as the first‐line therapy.

### 1.2 Outcomes

The efficacy assessment included progression‐free survival (PFS), overall response rate (ORR), and disease control rate (DCR). PFS was defined as the time from the first administration of eribulin to PD, according to RECIST version 1.1 or death from any cause. ORR was calculated as the proportion of patients with complete or partial response (CR or PR) while DCR was the sum of CR, PR, and stable disease (SD). Adverse events (AEs) were assessed according to the NCI Common Terminology Standard for Adverse Events (CTCAE5.0).

### 1.3 Statistical methods

Descriptive statistics were used to summarize patents' characteristics and safety data. Kaplan–Meier (K‐M) curves were utilized to illustrate PFS along with the corresponding 95% confidence intervals (95% CI), while the log‐rank tests were used to assess differences between groups. Additionally, univariate and multi‐factor analyses were performed to identify factors related to the effectiveness of eribulin; patients were categorized by the following criteria, line of therapy at the initiation of eribulin (≤2‐line vs. >2‐line), molecular typing (TNBC vs. non‐TNBC), presence of visceral metastasis (with or without), liver metastasis (with or without), number of metastases (≤2 vs. >2), combination therapy (yes or no), and ECOG PS (≤1 vs. 2). Variables with *p* < 0.05 were included in multivariate Cox regression analysis. All statistical analyses were performed using IBM SPSS26.0.

## 2 RESULTS

### 2.1 Patient characteristics

Between June 1, 2022, and December 31, 2022, a total of 134 female patients with ABC were enrolled. The baseline characteristics of patients are summarized in Table 1. The median age was 50 years (range, 22–70 years). Among the patients, 81 (60.4%) patients were HR‐positive and 20 (14.9%) patients were HER2 (+). The distribution of molecular types was as follows: 47 (35.1%) TNBC, 20 (14.9%) HER2 (+), and 67 (50.0%) HR+/HER2(−). Most (114/134, 85.1%) patients had visceral metastasis. Eighty (59.7%) patients had ≤2 metastatic sites, while the remaining had 3 or more metastatic sites. The most common metastatic sites were the liver (84 patients, 62.7%), lung (68 patients, 50.7%), bone (63 patients, 47.0%), and brain (17 patients, 12.7%). Other less frequent metastatic sites included the pleural, chest wall, contralateral breast, adrenal gland, spleen, pancreas, and bone marrow. Eribulin was used as a first‐, second‐, third‐, and subsequent‐line (≥3) chemotherapy agent in 22 (16.4%), 32 (23.9%), 20 (14.9%), and 60 (44.8%) patients, respectively. Fifty‐four (40.3%) had received up to two previous treatment lines, while 80 (59.7%) had received three or more previous treatment lines. The most frequently used chemotherapeutic agents in combination with eribulin (46/134, 34.3%) were carboplatin (29/134, 21.6%), capecitabine (13/134, 9.7%), platinum (1/134, 0.7%), gemcitabine (1/134, 0.75%), tegafur (1/134, 0.7%), and liposome adriamycin (1/134, 0.7%). Among the HER2 (+) patients, 7 (5.2%) patients received eribulin monotherapy due to resistance to anti‐HER2 therapy, while the remainder received eribulin received eribulin combined with anti‐HER2 therapy including pyrotinib (6/134, 4.5%), neratinib (2/134, 1.5%), and dual anti‐HER2 therapy (5/134, 3.7%). Additionally, 12 patients were treated with eribulin plus immune checkpoint inhibitors (ICIs), such as programmed cell death protein 1 (PD‐1) or PD‐1 ligand (PD‐L1) inhibitors, and 4 patients received eribulin plus carboplatin/capecitabine plus a PD‐1 inhibitor. Furthermore, 11 patients received eribulin in combination with anti‐angiogenesis agents (bevacizumab or anlotinib), and 2 patients received combination therapy of eribulin, anlotinib, and a PD‐1 inhibitor.

**TABLE 1:** Demographics and clinical characteristics of patients.

Characteristics	Patients	(%)
Age		
<50	89	66.4
≥50	45	33.6
Molecular typing		
HR(+)/Her2(–)	67	50.5
Her2(+)	20	14.9
TNBC	47	35.1
ER		
Positive	73	54.5
Negative	61	45.5
PR		
Positive	65	48.5
Negative	69	51.5
Her2		
Positive	20	14.9
Negative	114	85.1
Visceral metastases		
Yes	114	85.1
No	20	14.9
Number of metastases sites		
≤2	80	59.7
≥3	54	40.3
Liver metastases		
Positive	84	62.7
Negative	50	37.3
Lung metastases		
Positive	68	50.7
Negative	66	49.3
Bone metastases		
Positive	63	47
Negative	71	53
Brain metastases		
Positive	17	12.7
Negative	117	87.3
Number of eribulin therapy lines		
1‐line	22	16.4
2‐line	32	23.9
3‐line	20	14.9
≥4‐line	60	44.8
Combine other drugs		
No	46	34.3
Carboplatin	29	21.6
Capecitabine	13	9.7
Platinum	1	0.7
Gemcitabine	1	0.7
Tegafur	1	0.7
Liposome adriamycin	1	0.7
Anti‐HER2 agents	13	9.7
ICIs	12	9.0
Carboplatin/capecitabine + ICIs	4	3.0
Anti‐angiogenesis agents	11	8.3
Anti‐angiogenesis agents + a PD‐1 inhibitor	2	1.5

### 2.2 Efficacy

**FIGURE 1:**
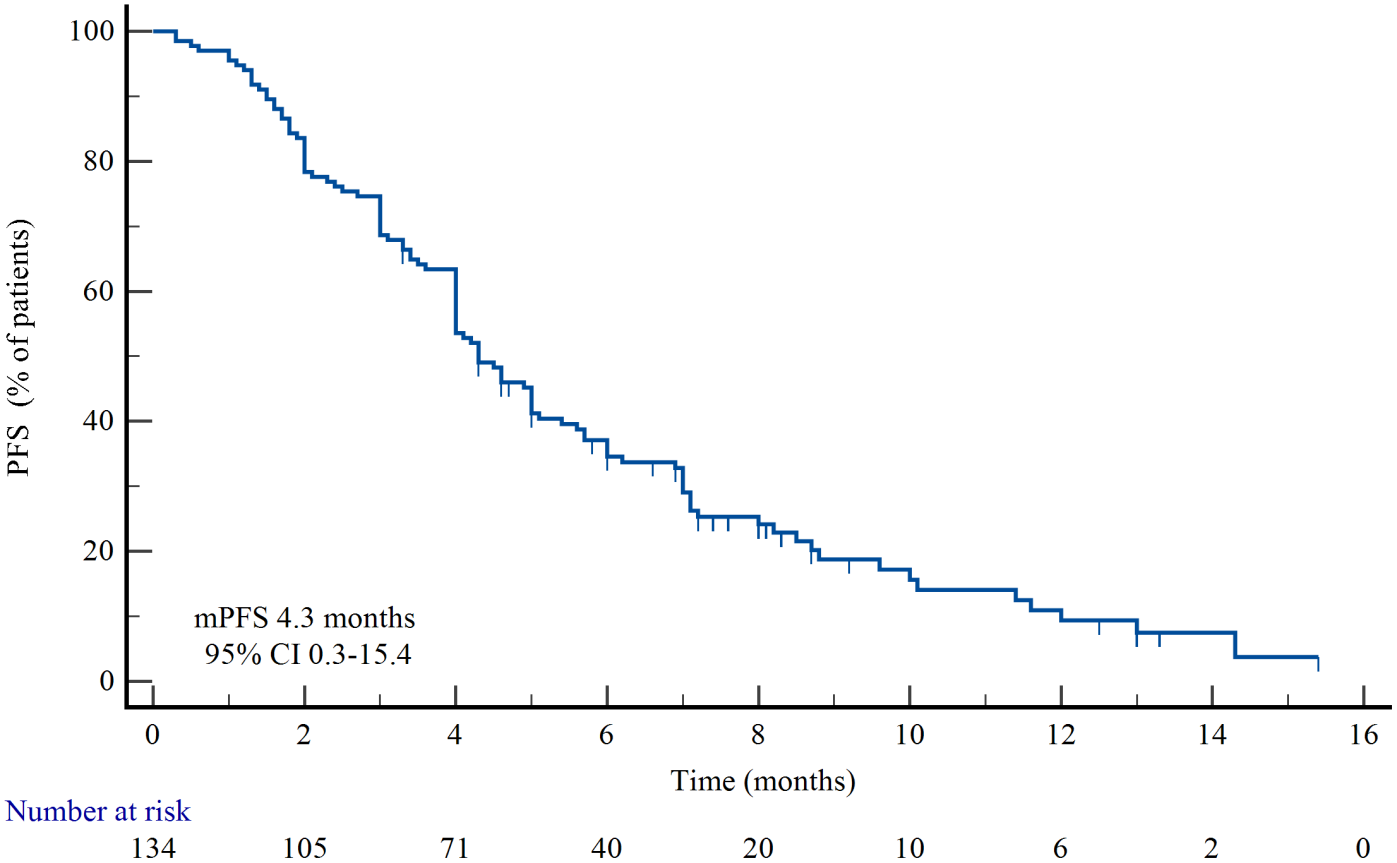
Progression‐free survival in ABC patients receiving eribulin.

The median number of cycles of eribulin treatment was 5 (1–14). As of data cutoff date on June 30, 2023, the median progression‐free survival (mPFS) was 4.3 months (95% CI: 0.3–15.4) for overall population (Figure 1). The best tumor response included CR in 1 (0.7%) patient, PR in 42 (31.3%) patients, SD in 63 (47.0%) patients, and PD in 28 (20.9%) patients. The ORR was 32.1% (43/134), and DCR was 79.1% (106/134) (Table 2).

**TABLE 2:** Overall efficacy evaluation all of patients.

Best response	*N* (%)
CR	1 (0.7)
PR	42 (31.3)
SD	63 (47.0)
PD	28 (20.9)
ORR	43 (32.1)
DCR	106 (79.1)

### 2.3 Subgroup analysis

#### 2.3.1 Line of therapy at initiation of eribulin

**FIGURE 2:**
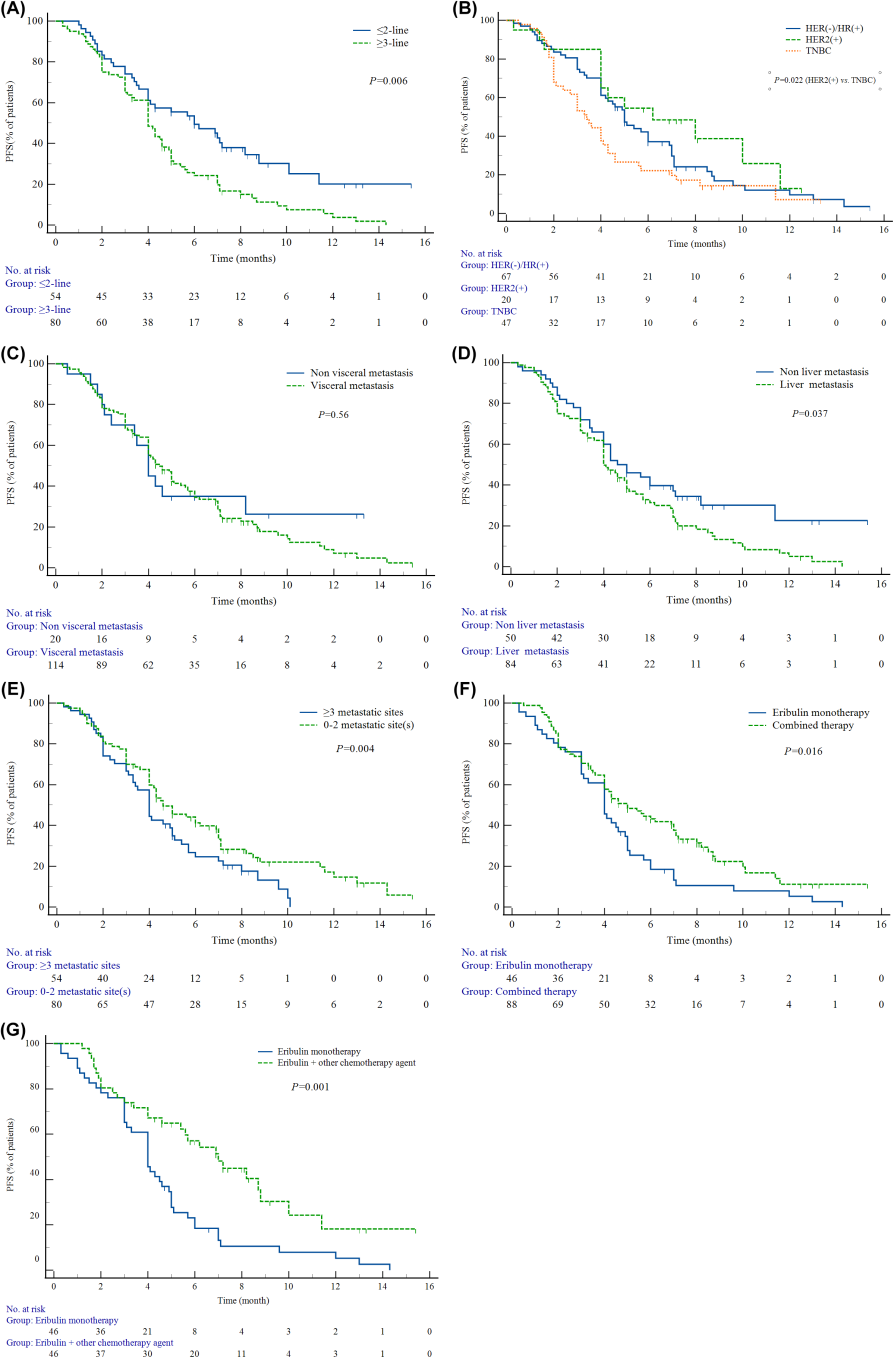
Kaplan–Meier curves for PFS according to potential predictive factors: (A) Line of therapy at initiation of eribulin; (B) Molecular type; (C) Visceral metastases; (D) Liver metastases; (E) Number of metastatic sites; (F) eribulin monotherapy or combine therapy; (G) whether or not combine chemotherapy drug.

The ORR in patients who received eribulin as 1–2 lines and ≥3‐line treatment were 54.0% (27/50) and 20% (16/80), respectively, *p <* 0.001. The mPFS of eribulin earlier lines group was significantly longer than eribulin ≥3‐line group (6.0 months [95% CI: 3.2–8.8] vs. 4.0 months [95% CI: 3.4–4.6], *p* = 0.006) (Figure 2A).

### 2.4 Molecular typing

The mPFS in TNBC, HER2(+), and HER2(−)HR(+) subgroups were 3.4 (95% CI: 2.7–4.1), 6.2 (95% CI: 2.3–10.1), and 5.0 months (95% CI: 4.1–5.9). Pairwise comparison showed that the mPFS difference between the TNBC and the HER2(+) group was statistically significant (*p* = 0.022), while the other groups had no statistically difference (Figure 2B).

### 2.5 Burden of disease

The mPFS benefit for patients with visceral metastasis was slightly poorer than their counterparts without visceral metastasis (4.0 months [95% CI: 3.3–4.7] vs. 4.5 months [95% CI: 3.7–5.2]), (*p* = 0.56) (Figure 2C). Further analysis indicated that liver metastasis and multiple metastases (>2 metastatic sites) were correlated to miserable prognosis. Patients without liver metastasis or with ≤2 metastatic sites showed a significantly longer mPFS than those with risk factors mentioned above (4.6 months [95% CI: 3.2–5.9] vs. 4.0 months [95% CI 3.4–4.5], *p* = 0.037; 4.6 months [95% CI: 3.5–5.6] vs. 4.0 months [95% CI 3.5–4.5], *p* = 0.004) (Figure 2D,E).

### 2.6 Combination therapy

The best tumor response between eribulin monotherapy and combination therapy group was shown in Table 3. Compared with monotherapy group, the combination therapy group led to significantly more PFS benefit and better ORR. The mPFS and ORR were 5.0 months (95% CI 3.6–6.3) vs. 4.0 months (95% CI: 3.3–4.7) (*p* = 0.016), and 48.7% (43/88) versus 19.6% (9/46) (*p* = 0.025), respectively (Figure 2F). Further analysis was conducted in patients who received eribulin monotherapy compared to those who received eribulin plus other chemotherapy agent, the ORR was 47.8% (22/46) versus 19.6% (9/46) (*p* = 0.004), and the mPFS was 7.0 months (95% CI: 5.1–9.0) versus 4.0 months (95% CI: 3.3–4.7) (*p* = 0.001) (Figure 2G).

**TABLE 3:** Best tumor response between eribulin monotherapy and combination therapy group.

Best response	Combine therapy group (%)	Eribulin monotherapy group (%)
CR	1 (1.1)	0 (0)
PR	33 (37.5)	9 (19.6)
SD	35 (39.8)	28 (60.7)
PD	19 (21.6)	9 (19.6)
ORR	43 (48.9)	9 (19.6)

### 2.7 Univariate and multivariate analyses for the predictors of PFS

Univariate analysis revealed that factors including line of therapy at initiation of eribulin, molecular type, liver metastasis, multiple metastases, combination therapy, and ECOG PS were significantly clinical variables associated with PFS when patients received eribulin therapy. Multivariate Cox regression analysis indicated that patients with non‐TNBC, eribulin as ≤2‐line treatment and eribulin combination therapy showed a significantly longer PFS than patients with TNBC, eribulin as >2‐line treatment and eribulin monotherapy. The other variables analyzed did not reach prognostic significance for PFS (Table 4).

**TABLE 4:** Univariate and multivariate multi‐factor analysis analyses for the predictors of PFS.

Prognostic factor	Univariate			Multivariate		
	HR	95% CI	*p*	HR	95% CI	*p*
Line of therapy at initiation of eribulin	1.83	1.24–2.69	0.02	1.844	1.141–2.982	0.013
Molecular typing	1.67	1.09–2.60		2.424	1.552–3.784	<0.001
Visceral metastasis	1.17	0.68–2.02	0.56	–	–	–
Liver metastasis	1.52	1.03–2.24		0.707	0.444–1.131	0.148
Number of metastatic sites	0.65	0.4338–0.99		0.846	0.555–1.291	0.438
Use of combination therapy with other drugs	2.28	1.41–3.69		1.317	1.047–2.050	0.043
ECOG performance statue	2.08	1.01–4.24	0.02	0.606	0.339–1.085	0.092

### 2.8 Safety

The most common AEs in this study were hematologic toxicities, including neutropenia (66.42%), leukopenia (20.90%), thrombocytopenia (12.69%), and anemia (5.97%). The most common treatment‐related AEs (TRAEs) were also hematologic toxicities, including neutropenia (33.58%), leukopenia (11.94%), and thrombocytopenia (4.48%). No treatment‐related death was recorded. Non‐hematological AEs were usually grade 1 or 2, including nausea, fatigue, alopecia, vomiting, peripheral neuropathy, headache, dizziness, diarrhea, constipation, abdominal pain, myalgia, decreased appetite, weight loss, oral mucositis, and hand–foot syndrome. Grade 3 or 4 non‐hematological AEs included peripheral neuropathy (4.48%) and diarrhea (1.49%) (Table 5).

**TABLE 5:** Adverse events.

AEs	All grade	A %	Grade 3/4 (%)
Neutropenia	89	66.42	33.58
Leukopenia	28	20.9	11.94
ALT increased	22	16.42	–
AST increased	18	13.43	–
Thrombocytopenia	17	12.69	4.48
Anemia	8	5.97	5.22
Blood bilirubin increased	8	5.97	–
LDH increased	3	2.24	–
Dyslipidemia	6	4.48	–
Fatigue	69	51.49	–
Alopecia	62	46.27	–
Nausea	53	39.55	–
Peripheral neuropathy	33	24.63	4.48
Anorexia	25	18.66	–
Diarrhea	19	14.18	1.49
Vomiting	18	13.43	–
Stomachache	17	12.69	–
Constipation	16	11.94	–
Headache	15	11.19	–
Oral mucositis	15	11.19	–
Arthritis/Myalgia	15	11.19	–
Fever	14	10.45	–
Lose weight	10	7.46	–
Hand‐foot syndrome	9	6.72	–
Rash	5	3.73	–
Dizzy	5	3.73	–
Hypokalemia	4	2.99	–

## 3 DISCUSSION

To further evaluate the efficacy and safety of eribulin in breast cancer, this prospective real‐world study enrolled 134 patients with ABC. The mPFS for all patients was 4.3 months, which was consistent with previous phase III clinical trials with larger sample size (mPFS: 3.7–4.2 months)^12^, ^13^, ^22^ and real‐world studies (mPFS: 3.61–5.06 months).^23^, ^24^, ^25^, ^26^ The ORR in our study was 32.1%, which was higher than those in EMBRAC study (12.0%) and Study 301 (11.0%).^12^, ^13^ The higher ORR in this study may be attributed to approximately 66% of patients receiving combination therapy, which likely improved the antitumor activity compared with eribulin monotherapy. Combination therapy with eribulin plus trastuzumab or pertuzumab in HER‐2(+) ABC patients resulted in an ORR of 34.8%.^27^ Another phase II clinical trial, the ORR of combining eribulin with gemcitabine reported an ORR of 37.3%.^28^

The study findings also indicated that eribulin treatment was more effective when used in earlier lines of therapy. The ORR of eribulin as first‐ or second‐line treatment (54.0%) was significantly better than that eribulin as ≥3 lines treatment (20.0%). Furthermore, patients receiving eribulin as the first‐ or second‐ line treatment had significantly longer mPFS compared to those receiving eribulin as ≥3‐line treatment (mPFS: 6.9 months vs. 4.0 months, respectively). Interestingly, the OS improvement with eribulin in the routine clinical practice appeared to be more notable than that observed in clinical trials. In the first real‐world, community‐based study of mTNBC conducted in the United States, 252 subjects were divided into two groups based on their prior systemic treatment lines: an early use group (≤2 lines) and a late use group (≥3 lines). In the early use group, the mOS was reported to be 23.0 months, which was obviously longer than the mOS reported in EMBRACE and Study‐301(13.1 months and 14.4 months, respectively) trials although the data from different studies could not be compared directly. For patients in the late‐line use group, who had more metastatic sites and received more previous systemic treatments, the mOS was 14.7 months.^12^, ^13^, ^29^ Another multicenter retrospective analysis showed that a significantly better mOS for patients with ≤2 previous chemotherapy lines was compared with those with >2 previous chemotherapy lines (328 vs. 264 days),^30^ Oruc et al. investigated the factors influencing the outcomes of eribulin treatment in 80 patients with MBC who had experienced 3–10 prior lines of treatment. Their multivariate COX analysis revealed that previous systemic treatment line was an independent prognostic factor affecting PFS. The mPFS in patients with three prior lines of treatment was 8.6 months, compared to 4.6 months in those >3 prior lines of treatment.^31^

It is well‐known that molecular typing is an important prognostic factor in ABC. Two phase II studies indicated that combing eribulin with HER‐2‐targeted agents in HER2(+) ABC could result in a PFS of about 9.2–11.6 months with tolerable toxicity.^20^, ^32^ However, patients with HER2(+) in our study had an obviously inferior mPFS (6.2 months) compared with those reported in previous studies, which may be attributed to that most of HER2(+) patients were heavily pretreated and received eribulin monotherapy. A recent real‐world study from United Kingdom revealed that patients with TNBC had a significantly worse prognosis than HER2(+)/ER(+) patients when treated with eribulin.^30^ The expression of hormone receptors is also an independent factor affecting PFS of patients with ABC. Rossi et al showed that HR(+) patients had almost twice as long PFS as HR(−) patients (mPFS: 2.9 vs. 1.4 months, *p* = 0.0051).^33^ Sirven et al. demonstrated that patients with TNBC had significantly shorter PFS than those with HR‐positive tumors (mPFS: 3.3 vs. 4.5 months).^23^ Similar results were found in another real‐world study (TNBC vs. HR(+) mPFS =3.4 vs. 2.0 months, *p* = 0.003).^33^ In our study, there was significant difference in PFS between the HER2+ and TNBC groups (mPFS = 6.2 vs. 3.4 months, *p* = 0.022), while the difference between TNBC and HER2(−)/ HR(+), or between HER2(+) and HER2(−)/ HR(+) were not statistically significant. Multivariate COX analysis demonstrated that TNBC was one of adverse prognostic factors for PFS of eribulin therapy.

In our study visceral metastasis, liver metastasis and multiple metastases were not key prognostic factors for PFS of patients with MBC. Some studies have found that the development of visceral metastasis usually predicts poor survival outcomes, especially for those with liver and/or lung metastases.^34^, ^35^ Dell'Ova et al. found that pulmonary metastasis (HR = 1.53 [95% CI: 1.16–2.02]) was one of the independent prognostic factors affecting TTF in ABC patients treated with eribulin.^36^ Similarly, an observational study in Japan showed that liver metastasis resulted a poor TTF in HER2(−) ABC.^37^ The result of our study showed that patients with or without visceral metastasis had no significant difference in mPFS benefit (4.0 [95% CI: 3.3–4.7] vs. 4.5 months [95% CI: 3.7–5.2], *p* = 0.56), which may be partly attributed to the lower proportion of enrolled patients without visceral metastasis (Figure 2C). Although univariate analysis showed that patients without liver metastasis had a longer mPFS than that of patients with liver metastasis (mPFS: 4.6 months [95% CI: 3.2–5.9] vs. 4.0 months [95% CI 3.4–4.5]) (Figure 2D). Multivariate Cox analysis found that liver metastasis was not an independent significant prognostic factor for PFS (Table 4). De Sanctis et al. found that the number of metastatic lesions was negatively correlated with PFS in the treatment of eribulin.^38^ The results of a multicenter retrospective study showed that multiple metastases were correlated with poor PFS, patients with 1–2 metastatic sites had a mPFS of 5.3 months while the mPFS of those with ≥3 metastatic sites was 3.6 months (*p* = 0.023).^39^ In our study, patients with ≤2 metastatic sites also achieved a mPFS of 4.6 months, significantly longer than that in patients with ≥3 metastatic sites (4.0 months, *p* = 0.044) (Figure 2E). However, multivariate Cox regression analysis indicated that the number of lesions did not reach prognostic significance for PFS.

The investigation of eribulin combination therapy is attracting increasing attention. In the ERIGE study, eribulin plus gemcitabine yield an ORR of 37.3% in the treatment of TNBC. The mPFS and mOS were 5.1 and 14.5 months, respectively. Subgroup analysis showed that BRCA wild‐type patients had better curative effect.^28^ A similar result was observed in our study, patients who received eribulin plus other chemotherapy agent had a better mPFS and ORR compared to those who received eribulin monotherapy (ORR: 47.8% vs. 19.6% *p* = 0.004, mPFS: 7.0 months (95% CI: 5.1–9.0) vs. 4.0 months (95% CI: 3.3–4.7), *p* = 0.001) (Figure 2G). In a single‐arm, multicenter, phase II study, the combination of eribulin and trastuzumab achieved an ORR of 71.2% in the first‐line treatment of HER2(+) MBC, and the mPFS was 11.6 months.^32^ The JBCRG‐M03 study indicated that the treatment regimen containing eribulin, pertuzumab, and trastuzumab was effective and generally well tolerated in the first‐ or second‐line treatment of HER2(+) ABC with a mPFS of 9.2 months (95% CI: 7.0–11.4 months).^20^ Based on this study, a phase III study evaluating the triple combination of eribulin, pertuzumab, and trastuzumab in patients with Her2(+) ABC has been carried out.^21^ A phase II study aimed to evaluate the efficacy and safety of eribulin plus anlotinib in patients with ABC; the mPFS of the experimental group and the control group was 5.1 versus 3.5 months, respectively (HR = 0.56, 95% CI: 0.32–0.98).^40^ In our study, 88 patients received eribulin combination therapy, and the mPFS of the eribulin monotherapy and combination therapy was 4.0 and 5.0 months, respectively (Figure 2F). Multivariate Cox regression analysis found that eribulin combination therapy is a predictor of better specific PFS.

Many other studies have explored the relationship between ECOG PS score and the efficiency of eribulin. Zhao et al. found that patients with different performance status had similar PFS; the difference of mPFS between ECOG 0–1 and ECOG 2 group was not significant.^39^ Conversely, De Sanctis et al. confirmed that a worse ECOG PS was related to a worse PFS.^38^ In our multivariate Cox regression analysis, there was no significant difference in PFS according to performance status (ECOG PS 0–1 vs. ECOG PS 2).

Several retrospective studies have demonstrated the safety of eribulin in breast cancer. The most common toxicities (all grades) included fatigue, nausea, neutropenia, and peripheral neuropathy, with neutropenia being the most common high grade (≥3) AE.^37^, ^41^ Although the majority of patients in our study received the elibulin combination therapy, the overall toxicity was tolerable. Hematological toxicity was the most common AEs, with grade 3/4 neutropenia and leukopenia were recorded in 33.58% and 11.94% of patients, respectively. Fatigue and nausea were the most common non‐hematological toxicities. Other AEs included diarrhea, constipation, oral mucositis, and stomachache.

Our study has some limitations. Firstly, this study lacked the non‐eribulin treated patients for comparison and the sample size was limited. As a real‐world study, potential confounders may introduce bias to the results of the prognostic analysis. The evaluation of eribulin related AEs was partly based on subjective patient experience, such as peripheral neuropathy, hand and foot syndrome, nausea, and vomiting. In addition, some patients completed the treatment in the outpatient department, leading to missing data on some AEs may be missing. The OS data were not mature due to limited follow‐up time, and the follow‐up is still ongoing to provide more clinical data on eribulin in Chinese patients with ABC.

## 4 CONCLUSION

In conclusion, this study demonstrated the effectiveness and tolerability of eribulin in patients with ABC in real‐world setting. Non‐TNBC, eribulin as ≤2‐lines treatment and combination therapy were related to the PFS benefit of the eribulin therapy.

## AUTHOR CONTRIBUTIONS

**Lan Chen:** Formal analysis (equal); writing – original draft (lead). **Xi Yan:** Conceptualization (lead); methodology (equal). **Ting Luo:** Conceptualization (supporting); methodology (equal). **Tinglun Tian:** Data curation (equal); resources (equal). **Ping He:** Data curation (equal); resources (equal). **Xiaorong Zhong:** Data curation (equal); methodology (equal); resources (equal); writing – original draft (supporting).

## CONFLICT OF INTEREST STATEMENT

The authors have no conflicts of interest to declare.

## ETHICS STATEMENT

This study was approved by the Biomedical Research Ethics Committee of West China Hospital of Sichuan University as No. 2022(862). All patients volunteered to participate the study and given the informed consent.

## Data Availability

The data that support the findings of this study are not publicly available due to privacy reasons.
